# An adapted Coffey model for studying susceptibility losses in interacting magnetic nanoparticles

**DOI:** 10.3762/bjnano.6.223

**Published:** 2015-11-19

**Authors:** Mihaela Osaci, Matteo Cacciola

**Affiliations:** 1 “Politehnica” University of Timisoara, Department of Electrical Engineering and Industrial Informatics, Piata Victoriei Nr. 2, 300006 Timisoara, jud. Timis, Romania; 2 University “Mediterranea” of Reggio Calabria, DICEAM, Via Graziella Feo di Vito, I-89100 Reggio Calabria, Italy

**Keywords:** hyperthermia, magnetic nanoparticles, relaxation process, specific loss power, susceptibility losses

## Abstract

**Background:** Nanoparticles can be used in biomedical applications, such as contrast agents for magnetic resonance imaging, in tumor therapy or against cardiovascular diseases. Single-domain nanoparticles dissipate heat through susceptibility losses in two modes: Néel relaxation and Brownian relaxation.

**Results:** Since a consistent theory for the Néel relaxation time that is applicable to systems of interacting nanoparticles has not yet been developed, we adapted the Coffey theoretical model for the Néel relaxation time in external magnetic fields in order to consider local dipolar magnetic fields. Then, we obtained the effective relaxation time. The effective relaxation time is further used for obtaining values of specific loss power (SLP) through linear response theory (LRT). A comparative analysis between our model and the discrete orientation model, more often used in literature, and a comparison with experimental data from literature have been carried out, in order to choose the optimal magnetic parameters of a nanoparticle system.

**Conclusion:** In this way, we can study effects of the nanoparticle concentration on SLP in an acceptable range of frequencies and amplitudes of external magnetic fields for biomedical applications, especially for tumor therapy by magnetic hyperthermia.

## Introduction

Magnetic nanoparticles are important for applications in biomedicine, in particular for hyperthermia-based treatments. Recent medical researches show that the heat generation of iron oxide nanoparticles in an alternating magnetic field activates an immune system response to tumors [1]. In magnetic nanoparticle systems for hyperthermia applications, one major issue is to control the parameters of the system, particularly the specific loss power (SLP). SLP is defined as the electromagnetic power lost per nanofluid mass unit. SLP is expressed in watts per kilogram. Recent researches show that the heating process through hyperthermia with magnetic nanoparticles is strongly affected by the choice of some parameters such as the amplitude and the frequency of the external magnetic field [1–2]. Typical parameters of AC magnetic fields are frequencies between 100 and 500 kHz with amplitudes ≤30 kA/m [2–3]. A recent investigation in humans resulted in an upper limit of 4.85·10^8^ Am^−1^s^−1^ for the product of frequency and field strength per one hour of treatment [4]. The influence of the nanoparticle concentration is implicitly related to the magnetic dipolar interactions among the nanoparticles. In order to select an assembly of magnetic nanoparticles suitable for use in hyperthermia, it is of interest to establish the conditions providing a sufficiently high SLP in an alternating external magnetic field of moderate amplitude *H*_ext_ and frequency *f* [5]. The latest researches suggest that the so-called “hard-magnetic nanoparticles” are a more reliable basis for practical magnetic hyperthermia than small nanoparticles [6]. Usually, the magnetite nanoparticles used in hyperthermia have a diameter above 53 nm but below the diameter above which objects are no more considered to be “nano” (100 or 200 nm, according to the bibliographic sources). Actually, magnetite is considered the most favourable material in magnetic hyperthermia. At about 30 nm particle diameter the behaviour of magnetite nanoparticles changes from single-domain to multi-domain state [7], representing the critical dimension of magnetite-based nanoparticles. The tendency for agglomeration and sedimentation increases considerably with the transition from small nanoparticles to stable ferromagnetic single domain [4]. This leads to clogging of blood vessels. The displacement of the domain wall causes the reversal of the magnetization direction in multi-domain nanoparticles. In a single-domain nanoparticle, it is possible to see how the energy barriers, expressed as a function of the domain wall movement, are relatively small if compared with the reversal of the complete magnetic moment. Thus, multi-domain nanoparticles are magnetically “softer” than single-domain nanoparticles. Consequently, multi-domain nanoparticles exhibit a lower hysteresis loss than single-domain nanoparticles [4]. For both theoretical and experimental researches in this field, choosing the right parameters of superparamagnetic nanoparticle systems to control magnetic hyperthermia is an important task [3,8–15].

It is well known that the magnetic monodomain nanoparticles exhibit unstable behaviour of their magnetic moments due to the thermal agitation, i.e., the magnetic moment of the nanoparticle can randomly change direction under the influence of temperature. This is called superparamagnetic behaviour [15]. The typical time between two flips is called relaxation time, and the reversal process is called relaxation process. In nanofluids, the superparamagnetic nanoparticles have two associated relaxation processes: the Néel relaxation process and Brownian relaxation process. The former is a solid-state mechanism that occurs within the nanoparticle. It corresponds to a switching of the magnetic moment between two equilibrium positions. The latter is due to the physical rotation of nanoparticle within the colloidal solution. Changing in orientation of the magnetic moment of a nanoparticle causes changes in the orientation of its magnetization [16].

At the limit between hysteretic and superparamagnetic regime, under biomedical conditions of amplitude and frequency of the external magnetic field, susceptibility losses in magnetic colloids can be described by linear response theory (LRT) [3]. Original LRT has been applied to a non-interacting system of nanoparticles close to equilibrium in low and variable applied magnetic fields. LRT offers a relationship between the volumetric loss power of ferrofluids and their magnetic relaxation [3]:

[1]
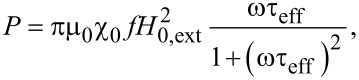


where *f* and *H*_0,ext_ are the frequency and magnitude of the applied field, respectively, μ_0_ is the magnetic permeability of free space, τ_eff_ is the average effective magnetic relaxation time of the nanoparticle system, and χ_0_ is the equilibrium average magnetic susceptibility of the nanoparticle assembly [3,9]:

[2]
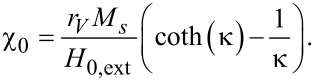


In Equation 2, *r_V_* is the volume fraction of the nanoparticles, with:

[3]
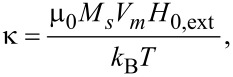


with *V_m_* being the average volume of nanoparticles. *k*_B_ the Boltzmann constant and *T* the temperature. The SLP describing the power per gram achievable in the magnetic material is given by:

[4]
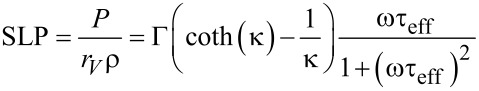


with

[5]
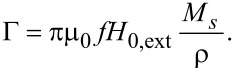


In Equation 4, ρ is the nanoparticle material density and κ is given by Equation 3. Equation 4 shows that SLP is a function of effective relaxation time, frequency and amplitude of the applied field. The effective relaxation time can be described as [2]:

[6]
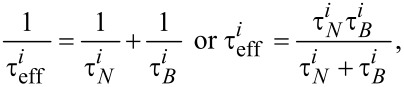


where 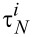 is the Néel relaxation time and 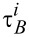 is the Brownian relaxation time. For spherical particles, Brownian relaxation time is usually described by [2]:

[7]
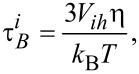


where *V_ih_* is the hydrodynamic volume and η is the coefficient of dynamic viscosity. Generally, the two processes are studied separately [2]. The relaxation process dominating the magnetic behaviour of the colloidal suspension is determined by the nanoparticle properties [16].

## Interacting colloidal magnetic nanoparticles

Single-domain nanoparticles have uniform magnetization state, regardless of the applied field. The magnetic moment can be formulated according to [14]:

[8]
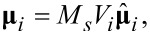


where *M_s_* is the spontaneous magnetization, *V_i_* is the particle volume and 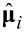 is the versor of the magnetic moments [16]. The contribution of the local magnetic field **H**_loc_ on each particle increases with the concentration of nanoparticles. **H**_loc_ results from a vectorial sum between the applied external magnetic field (**H**_ext_) and the internal dipolar magnetic field **H**_id_. The latter is given by the magnetic dipolar interactions among the nanoparticles:

[9]
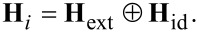


The magnetic moments of the *i*-th and *j*-th nanoparticle can be indicated with **μ***_i_* and **μ***_j_*, respectively, both with uniaxial anisotropy. Since nanoparticle *i* has a dipole–dipole magnetostatic interaction with all the other nanoparticles, the magnetic dipolar energy of the nanoparticle *i* and the local dipolar magnetic field acting on the nanoparticle *i* can be expressed as follows [17–18]:

[10]
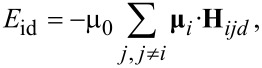


[11]
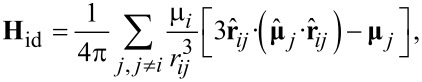


where *r_ij_* is the distance between the centres of those two nanoparticles, 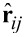 is the unit vector of the direction that connects the nanoparticles *i* and *j*, 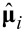 and 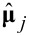 are the unit vectors of the magnetic moments of the nanoparticles *i* and *j*, respectively, and μ_0_ is the magnetic permeability of vacuum.

Due to clinical limitations on the amplitude of the external magnetic field **H**_ext_ [4,6,10], the anisotropy axes of the spherical nanoparticles are not perfectly aligned to the external magnetic field. Due to the internal dipolar magnetic field, a local magnetic field appears in nanoparticle according to Equation 9. This local magnetic field is, in general, not oriented along the anisotropy axis of each particle. For handling this situation, we adapted the Coffey analytical model [12] to the local magnetic fields according to Equations 9–11. Further descriptions are given in the following section.

## Néel relaxation time with the approximation of discrete orientation model

Usually, the thermal relaxation of nanoparticles subjected to an applied field is studied by the discrete orientations model of the magnetic moments [12,15]. In this model, we may assume that the magnetic moments **μ***_i_* are constrained to stable orientations along the local minimum of free energy, when the energy barriers are large in comparison with *k*_B_*T*. Due to thermal fluctuations, the time behaviour of **μ***_i_* is handled as a discrete Markov process, with *n_i_* (i.e., the number of particles in *i*-th orientation (*i* = 1, 2)) replacing the continuous distribution of orientation. For a large number *n* of nanoparticles, *n_i_* changes with time according to the following master equation [12,15]:

[12]
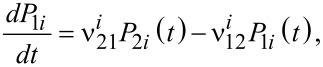


where *P*_1_*_i_*(*t*) is the probability of finding particles in the state 1 at the time *t*, and *P*_2_*_i_*(*t*) is the probability of finding particles in the state 2 at the time *t*. We denote the probability of transition, in units of time, from the state 1 to the state 2 with 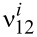, passing through the maximum. Moreover, let 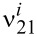 be the probability of transition, in time unit, from the state 2 to the state 1, passing through the maximum. The normalisation condition is *P_1i_*(*t*) + *P_2i_*(*t*) = 1. In Equation 12,


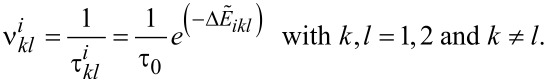


Here, 1/τ_0_ is the attempt frequency of magnetic reversal, considered constant with the value of 10^9^ s^−1^ [12,15]. Finally, consider the following relation:

[13]
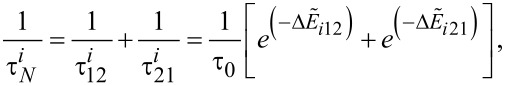


where 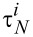 represents the Néel relaxation time for the particle *i* and 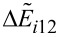, 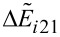 are the normalized energy barriers (over *k*_B_*T*) for the reorientation of the *i*-th magnetic moments. The Brownian relaxation time is given by Equation 7, and the effective relaxation time is given by Equation 6.

The problem of determining energy barriers in the systems with interactions is quite complex. Generally, in 3D space, the normalized (over *k*_B_*T*) free energy of the *i*-th spherical nanoparticle, subject to a local magnetic field [13], is:

[14]
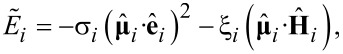


where ξ*_i_* = (μ_0_ *M_s_* *V_i_* *H_i_*)/(*k*_B_*T*), σ*_i_* = (*K_i_*_,eff_*V_i_*)/(*k*_B_*T*), and *K_i_*_,eff_ is the effective anisotropy constant of the *i*-th nanoparticle. Moreover, 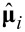 is the unit vector in the direction of magnetic moment of the *i*-th nanoparticle, 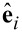 is the unit vector of the easy anisotropy axis of the *i*-th nanoparticle, and 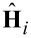 is the unit vector in the direction of the local magnetic field acting on the *i*-th nanoparticle.

Without losing the generality of the problem, it is possible to consider a magnetic field acting onto the *x*–*z* plane. Considering Equation 14, the normalized free energy of an *i*-th nanoparticle subject to a local magnetic field **H***_i_* applied with an angle ψ*_i_* to the easy axis is given by:

[15]
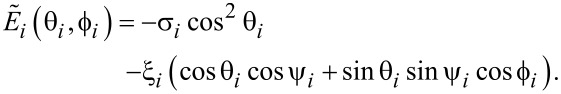


The polar axis is the easy anisotropy axis of the *i*-th nanoparticle, ψ*_i_* is the angle between **H***_i_* and the easy anisotropy axis of the *i*-th nanoparticle.

The stationary points of the normalized (over *k*_B_*T*) potential energy occur for 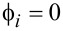 and 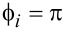. The stationary point for 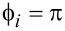 corresponds to a maximum of 
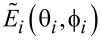
 in Equation 15 and, therefore, it is not of our interest [12]. The stationary point at 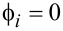 correspond to a saddle point of 
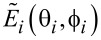
 at θ_0_*_i_*, and two minima at θ_1_*_i_* and θ_2_*_i_* for *h_i_* *< h_ic_*. Here, *h_ic_* is the critical value of *h* at which the normalized potential of Equation 15 loses its bistable behaviour [12]. In case of ψ*_i_* = 0, the magnetic moment of a given nanoparticle *i* can be in one of the two equilibrium states, with minimum energies determined by θ*_i_*_1_ = 0 and θ*_i_*_2_ = π. These minima are separated by the maximum. The normalized energy barriers for these re-orientations are:

[16]
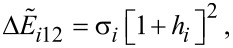


[17]
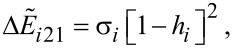


with *h_i_* = (μ_0_ *M_s_* *H_i_*)/(2*K_i_*_,eff_). In case of ψ*_i_* = π/2,

[18]
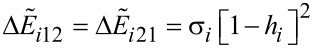


For other ψ*_i_* values, it is possible to use the Pfeiffer approximation [12,19] and the lower normalized energy barrier, in accordance with the following approximation:

[19]
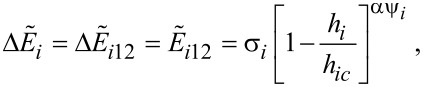


where αψ*_i_* = 0.86 + 1.14*g*ψ*_i_* and *h_ic_* = (cos*^2/3^*ψ*_i_* + sin*^2/3^*ψ*_i_*)*^−3/2^*.

## Adapted Coffey model for the Néel relaxation time of nanoparticles under oblique local magnetic fields

The original Coffey analytical model has been developed for Néel relaxation times under an oblique external magnetic field applied on a system of non-interacting nanoparticles [12–13]. Its analytical calculation is based on the Kramer theory [20] and is in agreement with the numerical calculation and experimental results [21]. This calculation shows the dependence of the relaxation time on the magnetic damping constant α. For the case of most ferromagnetic and ferrimagnetic nanoparticle systems, the magnetic damping constant α exhibits low values (α *<<* 1) [22]. In this section, we adapt the Coffey analytical model according to Equations 9–11. Under these conditions, the time relaxation relation, in case of an oblique magnetic field, is [12]:

[20]
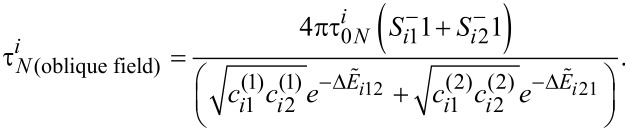


With 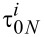 being the free diffusion magnetization time at low damping constants (α *<<* 1) [12]:

[21]
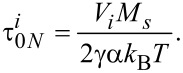


In Equation 21γ is the gyromagnetic ratio.

If ψ*_i_* is the angle between the local magnetic field **H***_i_* and the easy anisotropy axis of the *i*-th nanoparticle, θ*_i_* is the angle between the magnetic moment of the *i*-th nanoparticle and local magnetic field, *h_i_* = (μ_0_ *M_s_* *H_i_*)/(2*K_i_*_,eff_) and σ*_i_* = (*K_i_*_,eff_*V_i_*)/(*k*_B_*T*), then θ*_ip_* are the solutions of the following transcendental equation, with *p* = 1,2:

[22]
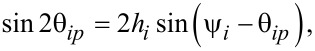


obtained by imposing

[23]
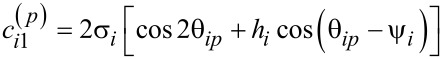


and

[24]
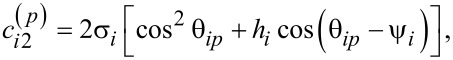


where *K_i_*_,eff_ being the effective anisotropy constant of the *i*-th nanoparticle.

In case of *h_i_* *< h_ic_*(ψ*_i_*) *<* 1 with *h_ic_* = (cos*^2/3^*ψ*_i_* + sin*^2/3^*ψ*_i_*)*^−3/2^* [12]:

[25]
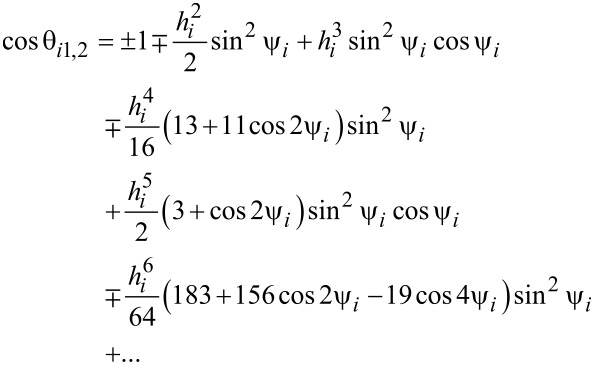


The normalized energy barriers for *i*-th magnetic moment reorientations 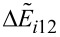, 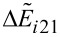 are:

[26]
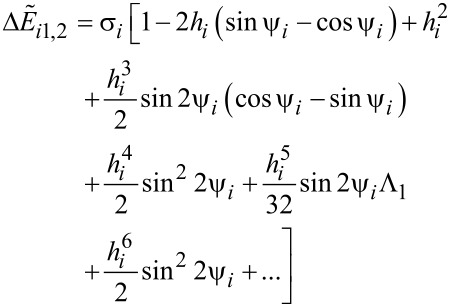


[27]
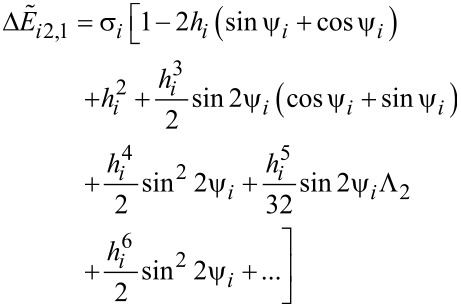


[28]
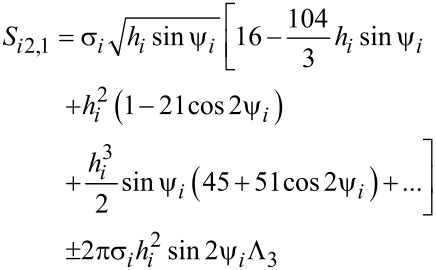


with


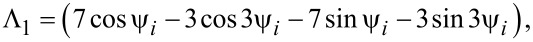



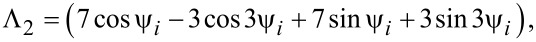


and


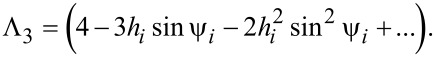


## Numerical simulations

We considered a system with spherical nanoparticles made of uncoated magnetite, with the following characteristics: density ρ = 5180 kg/m^3^ [3]; saturation magnetization *M_s_* = 4.46·10^5^ A/m [3]; uniaxial magnetic anisotropy with anisotropy constant *K*_eff_ = 25·10^3^ J/m^3^ [3]; random orientation of the anisotropy axis or anisotropy axis parallel with external magnetic field. We used an aqueous basic solution with a dynamic viscosity of 8.9·10^−4^ Pa·s, at the temperature *T* = 293 K. We applied a sinusoidal external magnetic field with an amplitude equal to 15 kA/m, at a frequency *f* = 300 kHz, typical for magnetic hyperthermia applications [10]. We point out that the model can be extended easily to the case of coated nanoparticles.

In terms of the arrangement of nanoparticles in three-dimensional space, we analysed two cases: a system with 1000 nanoparticles uniformly and randomly distributed, and a clustered subsystem of nanoparticles. We considered the case of cluster forming because nanoparticles aggregate and form clusters in real-world specimens. Simulated clusters contain 50 nanoparticles with random local distribution. We defined the local volume fraction *r_V_*_,loc_, i.e., the volume fraction of nanoparticles in the cluster. The global volume fraction *r_V_* is the volume fraction in the simulated specimen. In case of uniformly and randomly distributed particles, or the clustered subsystem, the nanoparticles have been located within a body-centered cubic lattice. Considering each lattice unit cell, the possible spatial locations where to place nanoparticles are the vertexes and the centre of each face of the lattice unit cell. We randomly selected suitable locations among all these possible positions, according to the ratio between the total volumes of nanoparticles and the whole volume of the cubic simulation box, i.e., the so-called volume fraction. Here, we applied periodic boundary conditions to our model, on each spatial direction [16]. The dipolar local magnetic field acting on the *i*-th nanoparticle is calculated with the Ewald summation method [23]. We inspected the case of random orientations of anisotropy axes as well as easy axes parallel to the external magnetic field. In both cases, we calculated τ*_N_* and τ*_eff_* with models described in the two previous sections. SPL is calculated with LRT theory [3], through the average effective relaxation time of interacting system.

In our figures, we used the following legend for our results: i) Results retrieved by the discrete orientation model are labelled as “with approximation-random” or “with approximation-parallel”. ii) Results obtained by the analytical Coffey model are labelled as “with analytical calculation-random” or “with analytical calculation-parallel”. In case of systems with uniform distribution of nanoparticles, SLP is calculated with Equaiton Equation 4. For local studies of clusters, we calculated SLP as a function of the volume fraction by the relation:

[29]
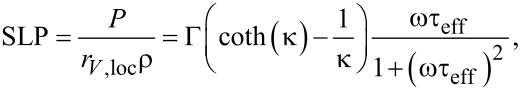


[30]
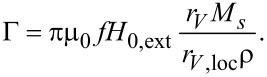


It should be noted that the volume fraction is a measure of the concentration of the nanoparticles within the colloidal solution. It is given by the ratio of the volume occupied by nanoparticles over the total volume of the solution. This means that the higher the volume fraction, the higher the concentration of the nanoparticles inside the solution. We extended this concept to clusters of nanoparticles, too.

## Results and Discussion

In this work, we considered monodisperse systems of nanoparticles, with diameters of 7 nm and 17 nm and a constant anisotropy, and we simulated for various nanoparticle volume fractions ranging between 0.01 and 0.24. But the model can easily be extended to nanoparticle systems with constant size and anisotropy distributions.

**Figure 1:**
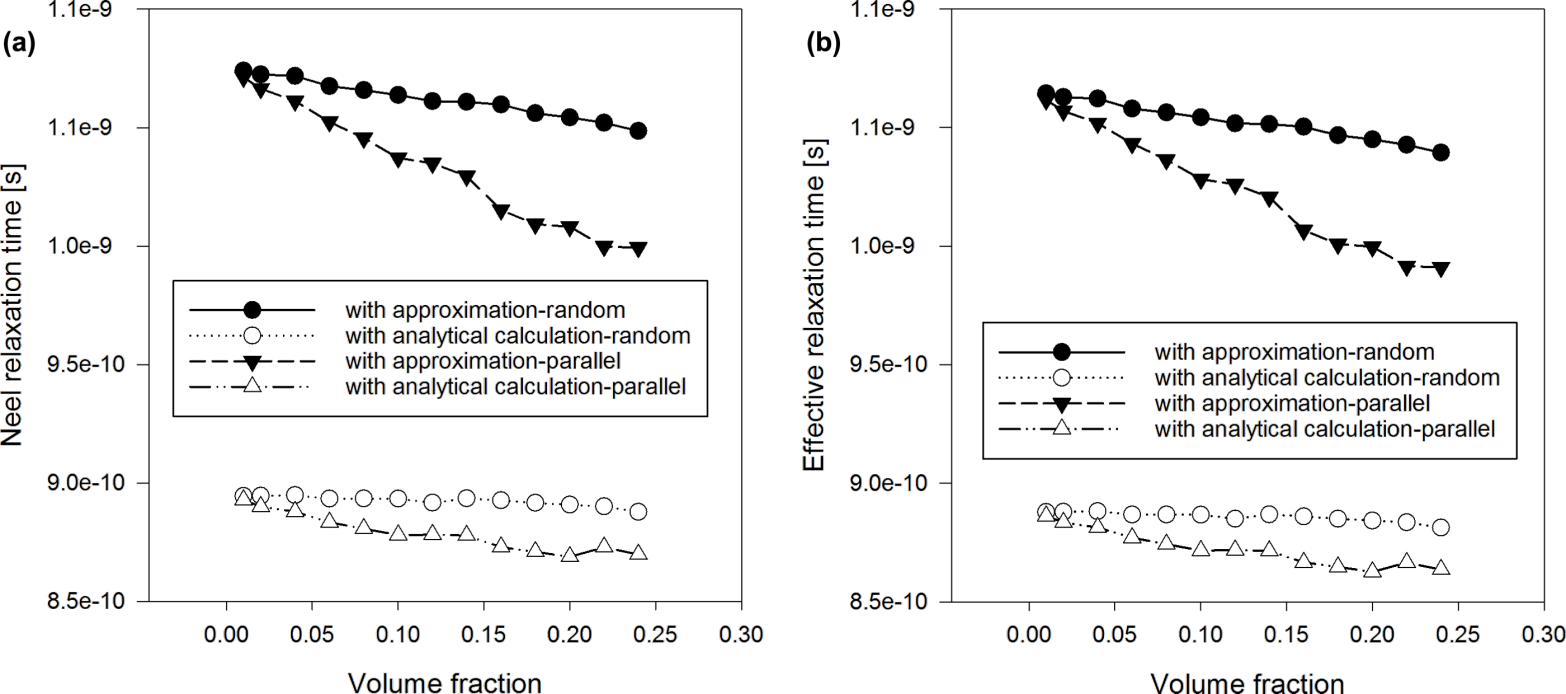
(a) Néel relaxation time and (b) effective relaxation time vs volume fraction of nanoparticles with diameter 7 nm.

**Figure 2:**
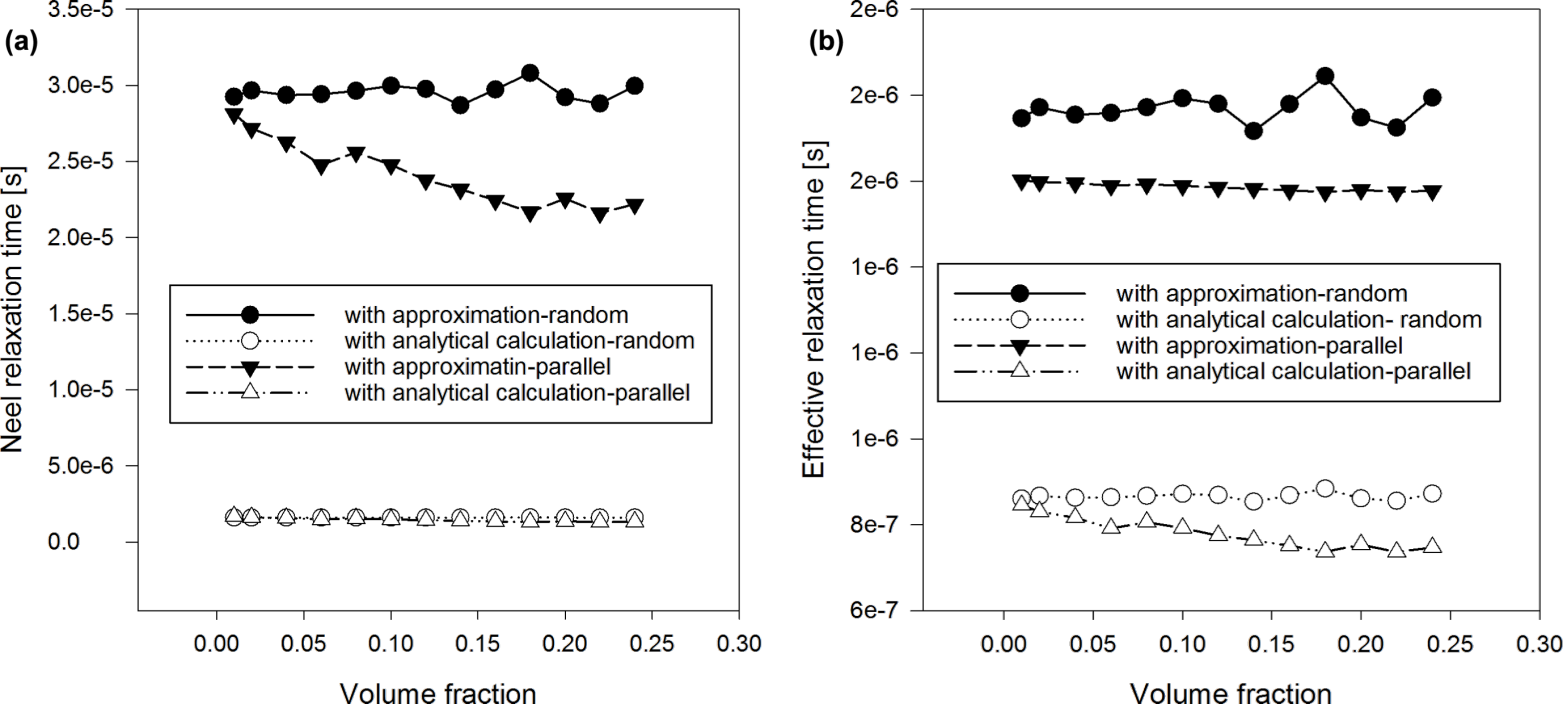
(a) Néel relaxation time and (b) effective relaxation time vs volume fraction of nanoparticles with diameter 17 nm.

**Figure 3:**
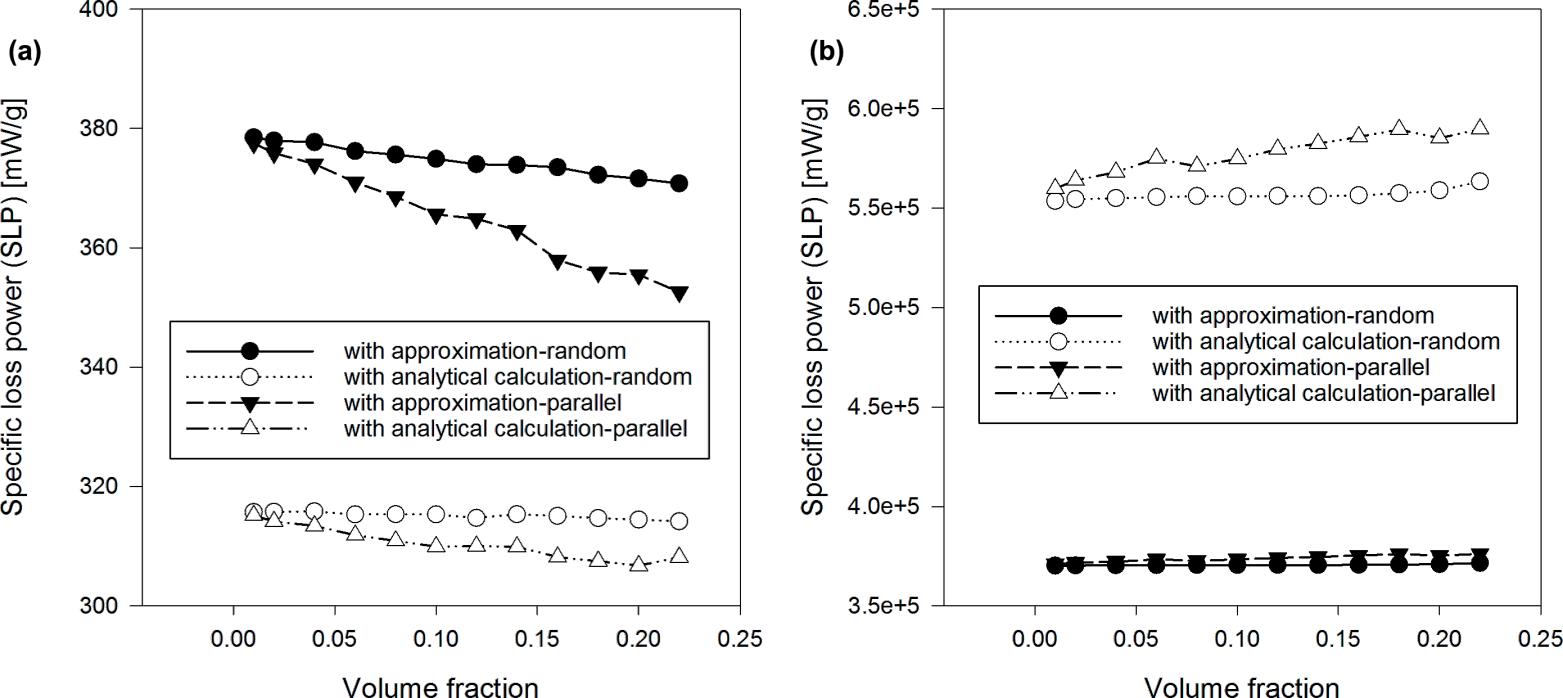
SLP vs volume fraction of nanoparticles: (a) diameter 7 nm, (b) diameter 17 nm.

**Figure 4:**
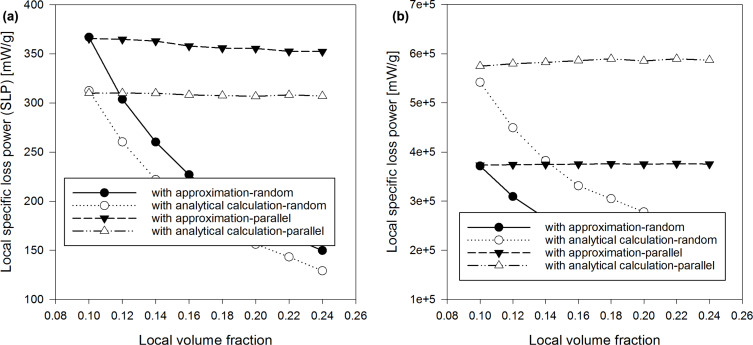
SLP vs local volume fraction in clusters of nanoparticles: (a) diameter 7 nm, (b) diameter 17 nm.

We calculated the average τ*_N_* (Figure 1 and Figure 2) by using the classical model from Equation 13 as well as the adapted Coffey analytical model from Equation 20. We carried out our calculations in case of randomly oriented anisotropy axes as well as with anisotropy axes parallel to the external magnetic field. In Equation 20, we used the pre-exponential factor τ_0_ = 10^−9^ s. Moreover, we simulated the trend of SLP respect to the global (Figure 3) and the local (Figure 4) volume fraction. For these latter studies, we have maintained the same peak value of the applied sinusoidal external magnetic field at 15 kA/m, fixing the frequency at 300 kHz, typical for magnetic hyperthermia applications [10]. Results obtained by the discrete orientation model are marked in figures by “with approximation” and results obtained by adapted Coffey analytical model by “with analytical calculation”.

**Figure 5:**
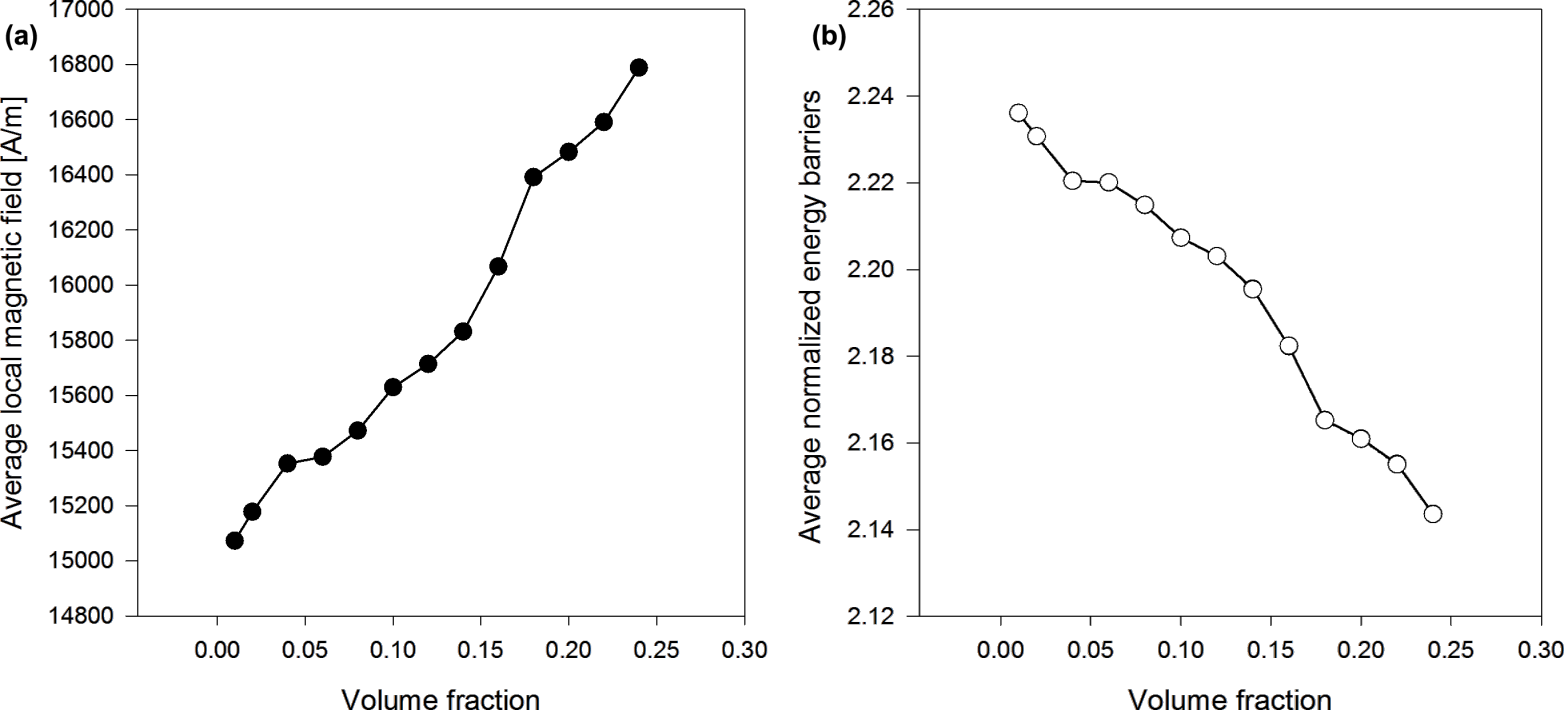
(a) Average magnetic field and (b) average normalized energy barriers vs volume fraction for nanoparticle system with 10 nm diameter.

The dependence of the average τ*_N_* and τ_eff_ on the volume fraction shows that they are higher in the discrete orientation model than in the adapted Coffey analytical model, at small concentrations of nanoparticles, when the particle diameter is 7 nm (Figure 1). This is even more the case when the nanoparticle diameter is 17 nm (Figure 2). As the concentration of nanoparticles increases, the difference between the approximate and analytical results decreases. In both cases, the Néel and the effective relaxation times decrease with increasing concentration. This behaviour is confirmed by the scientific literature, in theoretical as well as experimental works [7,10,21,24]. This is because the local magnetic field increases and energy barriers decrease as the concentration increases (Figure 5). Note also that the orientation of the anisotropy axes affects relaxation times. In the case of a random orientation, relaxation times are higher than in the case of parallel orientation. This effect is becoming more pronounced with increasing concentration of the nanoparticles. In Figure 1 and Figure 2, we can see that τ*_N_* and τ_eff_ are strongly affected by the size of nanoparticles, i.e., they strongly increase with increasing nanoparticle diameters.

With regard to the dependence of the SLP (calculated by the LRT with Equation 4 for a system of nanoparticles and with Equation 29 for clusters) we note the following: In case of an uniform distribution of the nanoparticles, SLP increases with increasing concentration for high diameters (17 nm) and decreases with increasing concentration for small diameters (7 nm). Please, refer to Figure 3. Figure 3 shows a difference between the SLP calculated with the approximated Equation 13 and the Néel component assessed by the analytical Equation 20. Scientific literature deals with either an increase or a decrease of the SLP with increasing nanoparticle concentration. Considering the nanoparticle system with 0.1 global volume fraction as well as clusters of 50 nanoparticles (with variations of the local concentration of clusters), the local SLP strongly decreases with an increasing nanoparticle concentration in clusters, in case of the anisotropy axis being randomly oriented with respect to the external magnetic field (Figure 4).

There is a big difference between the SLP assessed with the approximated average τ_eff_ and the corresponding SLP calculated with the actual average τ_eff_ using τ*_N_* from Equation 20 (Figure 3). The difference is more evident at high diameters of the nanoparticles. Moreover, we can see a strong influence of the orientation of the anisotropy axes. Please, note that the orientation of anisotropy axes affects the SLP. SLP is greater with random orientations than with parallel orientations for nanoparticles with small diameter (7 nm). The opposite occurs for high diameters of nanoparticles (17 nm). These effecs become larger when the concentration of nanoparticles increases. In clusters, SLP is lower with random orientations than with parallel orientations, for small as well as high diameters of nanoparticles (Figure 4).

**Figure 6:**
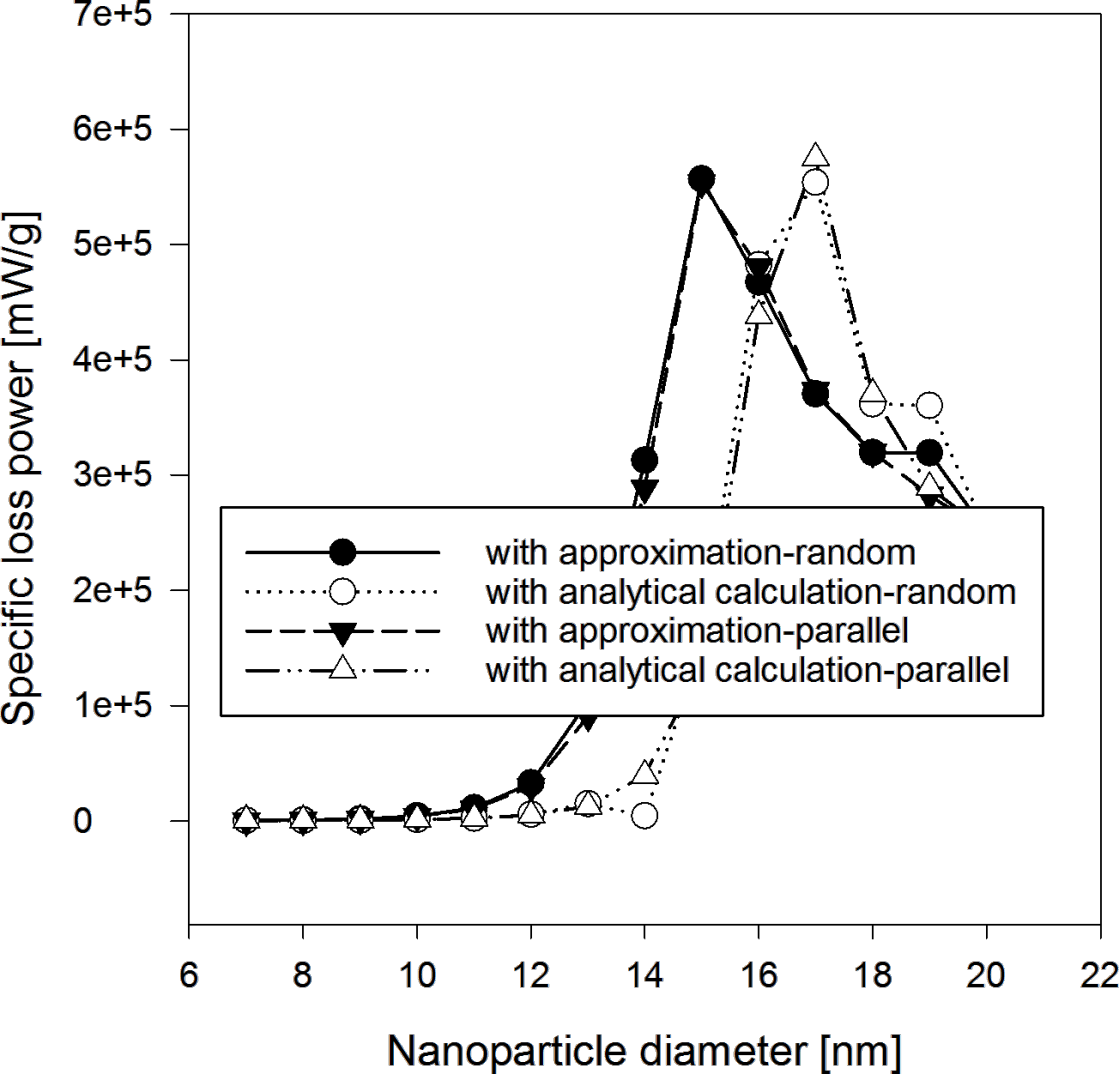
SLP vs diameter of the nanoparticles.

When simulating the SLP dependence on the nanoparticle size in a monodisperse system of uniformly distributed nanoparticles with constant particle-by-particle anisotropy, we found the presence of a maximum at the diameter of 17 nm (Figure 6) in the analytical calculation and at the diameter of 15 nm in the approximated calculation. We could see that this dependence is not influenced by assessing the τ*_N_* in case of small (*<*11 nm) and large radius (*>*19 nm). Note that this dependence is not influenced by the orientation of the axes of anisotropy. Calculation of SLP with numerical method, involving the analytical determination of τ*_N_* at oblique local magnetic field for 1000 nanoparticles, agrees with experimental data [25], for SLP measured for iron oxide (Table 1 and Table 2). Table 2 shows that the calculated SLP values determined by the common discrete orientation model are far from the measured results.

**Table 1:** Quantities used for evaluating modelled data with experimental results [25].

mean core diameter (nm)	hydrodynamic diameter (nm)	*r_V_*	*K_i_*_,eff_ (kJ/m^3^)	*H*_ext_ (kA/m)	*f* (kHz)	χ_0_

14 ± 0.21	26	0.0867	1.6·10^4^	24.5	400	12.31
12.8 ± 0.22	24.8	0.252	1.8·10^4^	24.5	400	7.98

**Table 2:** Experimentally obtained and numerically calculated (adapted Coffey method to local magnetic fields and common discrete orientation model for τ*_N_*) values of SLP.

experimental SLP (W/g)	discrete orientation SLP (W/g)	adapted Coffey SLP (W/g)

447	4204.553	471.523
200	2268.141	231.695

## Conclusion

In this paper, we adapted the Coffey analytical model to local magnetic fields. Then we compared this model with a common discrete orientation model. We studied how the concentration of nanoparticles and the orientations of anisotropy axes of nanoparticles influence the interactions among nanoparticles, in terms of relaxation times and SLP, when dealing with obliquely oriented local magnetic fields. The dependence of the average Néel and effective relaxation times on the volume fraction shows that the Néel relaxation time assessed by analytical calculation is higher than the Néel relaxation time approximately calculated, at low concentrations of nanoparticles. This difference is also confirmed by the SLP. The difference between the analytical and approximated results increases with the concentration of nanoparticles. In both cases, the Néel and effective relaxation times decrease with increasing concentration. Moreover, the Néel and effective relaxation times are strongly affected by the size of nanoparticles, i.e., they strongly increase with increasing nanoparticle diameters.

In case of an uniform distribution of nanoparticles, SLP increases with the concentration of nanoparticles for high radius of nanoparticles. In case of nanoparticle assemblies, SLP decreases with increasing concentration of clustered nanoparticles, when the local concentration varies. All these calculations are influenced by the orientation of anisotropy axes. In such systems, the SLP dependence on the nanoparticle size revealed the presence of a maximum at the diameter of 17 nm with analytical model, and 15 nm with approximated model.

Calculation of SLP with numerical the method, involving the analytical determination of τ*_N_* at oblique local magnetic field for 1000 nanoparticles, agrees with experimental data.

This adapted model can be used for small interacting nanoparticles in the linear response regime (low applied magnetic field) under the condition of *h_i_* *<* 0. The model can be easily extended to coated nanoparticles and to systems of nanoparticles with size and anisotropy constant distributions. Our studies can contribute to a better understanding of the susceptibility loss process and its biomedical implications, aiming to choose a suitable model for controlling this process in order to improve the therapeutic results.

## References

[1] Toraya-Brown S, Sheen M R, Zhang P, Chen L, Baird J R, Demidenko E, Turk M J, Hoopes P J, Conejo-Garcia J R, Fiering S (2014). Nanomedicine.

[2] Yelenich O, Solopan S, Kolodiazhnyi T, Tykhonenko Y, Tovstolytkin A, Belous A (2015). J Chem.

[3] Rosensweig R E (2002). J Magn Magn Mater.

[4] Dutz S, Hergt R (2014). Nanotechnology.

[5] Deatsch A E, Evans B A (2014). J Magn Magn Mater.

[6] Kashevsky B E, Kashevsky S B, Korenkov V S, Istomin Y P, Terpinskaya T I, Ulashchik V S (2015). J Magn Magn Mater.

[7] Heider F, Dunlop D J, Sugiura N (1987). Science.

[8] Coffey W T, Gregg P J, Kalmykov Y P, Prigogine I, Rice S (1992). On the theory of Debye and Nèel relaxation of single domain ferromagnetic particles. Advances in Chemical Physics.

[9] Ferguson R M, Minard K R, Khandhar A P, Krishnana K M (2011). Med Phys.

[10] Silva A C, Oliveira T R, Mamani J B, Malheiros S M, Malavolta L, Pavon L F, Sibov T T, Amaro E, Tannùs A, Vidoto E L (2011). Int J Nanomed.

[11] Hervault A, Thanh N T K (2014). Nanoscale.

[12] Coffey W T, Kalmykov Y P (2012). J Appl Phys.

[13] Coffey W T, Crothers D S F, Dormann J L, Geoghegan L J, Kalmykov Yu P, Waldron J T, Wickstead A W (1995). Phys Rev B.

[14] Haase C, Nowak U (2012). Phys Rev B.

[15] Kechrakos D, Sattler K (2010). Magnetic Nanoparticle Assemblies. Handbook of Nanophysics: Nanoparticles and Quantum Dots.

[16] Cacciola M, Osaci M (2015). IOP Conf Ser: Mater Sci Eng.

[17] Beke D L (1998). Cryst Res Technol.

[18] Mørup S, Hansen M F, Frandsen C (2010). Beilstein J Nanotechnol.

[19] Pfeiffer H (1990). Phys Status Solidi A.

[20] Hänggi P, Talkner P, Borkovec M (1990). Rev Mod Phys.

[21] Coffey W T, Crothers D S F, Dormann J L, Kalmykov Yu P, Kennedy E C, Wernsdorfer W (1998). Phys Rev Lett.

[22] Fannin P C, Malaescu I, Marin C N (2005). J Magn Magn Mater.

[23] Cline J I, Lorenz K T, Wade E A, Barr J W, Chandler D W (2001). J Chem Phys.

[24] Parvin K, Ma J, Ly J, Sun X C, Nikles D E, Sun K, Wang L M (2004). J Appl Phys.

[25] Gonzales-Weimuller M, Zaisberger M, Krishnan K M (2009). J Magn Magn Mater.

